# Using context mapping for planning implementation of movement behavior change in physiotherapy

**DOI:** 10.1186/s12913-025-13897-x

**Published:** 2025-12-22

**Authors:** Sofie van Rongen, Joan M. Dallinga, Anita Feleus, Ingrid Rosbergen, Alice Schut, Myrenka Bik, Petra Siemonsma, Sanne I. de Vries, Arlette E. Hesselink

**Affiliations:** 1 Research Group Healthy Lifestyle in a Supporting Environment, The Hague University of Applied Sciences, The Hague, The Netherlands; 2 Research Center Innovations in Care and Department of Physiotherapy, Rotterdam University of Applied Sciences, Rotterdam, The Netherlands; 3 Research group Self-Management in Physical Therapy and Human Movement Care, Department of Physical Therapy, Faculty of Health, University of Applied Sciences Leiden, Leiden, The Netherlands; 4 Department of Public Health and Primary Care, Health Campus the Hague, Leiden University Medical Centre, The Hague, The Netherlands

**Keywords:** Physical therapist, Implementation, Physical activity, Health promotion, Behaviour change

## Abstract

**Background:**

Physiotherapists often lack a comprehensive understanding of self-management and encounter challenges in providing self-management support. The Keep Moving Support Tool (KMST) was developed to support physiotherapists in their role as health promotors and to stimulate physical activity in patients with (risk of) chronic conditions. The KMST requires a change in workflow and behaviour of professionals, therefore it is crucial to meticulously identify barriers and facilitators during the pre-implementation stage to address them before the actual implementation. The aim of this study was to develop an implementation plan directed at the integration of the KMST within everyday practice in multiple physiotherapy clinics.

**Methods:**

A two-step research approach was applied using the Consolidated Framework for Implementation Research. First, a needs assessment was performed to identify barriers and facilitators for the implementation of the KMST. This process involved semi-structured interviews with four implementation experts with scientific knowledge and experience in the physiotherapeutic field and three context mapping sessions with four physiotherapists. Second, implementation strategies were selected and combined with their proposed operationalization (materials and actions) to address the barriers and facilitators.

**Results:**

The inventory of barriers and facilitators was grouped into five themes; (1) the KMST needs to be understood and appreciated, (2) physiotherapists need to feel and be competent to work with the KMST, (3) the KMST needs to be applied in physiotherapy clinics and implementation needs to be monitored, (4) several interest holders need to be involved, and (5) resources are necessary to facilitate implementation. Each theme included both barriers and facilitators. The developed implementation plan consists of various implementation strategies and practical materials and actions: develop communication materials and an e-learning to train physiotherapists how to apply the KMST, compose a core team of early adoptors and a learning community, align the KMST with the electronic patient file, monitor and evaluate application of the KMST.

**Conclusions:**

The combined data from the interviews and context mapping sessions helped to identify key factors that need to be addressed to enhance successful implementation of the KMST in daily practice of physiotherapy clinics. A future effect study should assess whether the implementation materials and actions effectively support physiotherapists in applying the KMST, and whether this leads to sustained physical activity among their patients.

**Supplementary Information:**

The online version contains supplementary material available at 10.1186/s12913-025-13897-x.

## Background

The urgency of sustained physical activity (PA) increases, as people are getting older and the number of people with one or more chronic diseases continues to increase [1–3]. Despite the well-known benefits of PA, only 43% of Dutch adults with a chronic health condition meets the *Physical Activity Guideline* [4]. For physiotherapists, health promotion, i.e. “the process of enabling people to increase control over, and to improve their health” [5], is an increasingly important task [6]. This requires a paradigm shift in the physiotherapist’s role in the treatment process, from a traditional role of the physiotherapist to one which includes person-centered guidance and self-management of sustained PA [7]. However, physiotherapists often lack a comprehensive understanding of self-management and encounter challenges in providing self-management support [8, 9]. Therefore, it is necessary to implement a new, person-centered behavior change method that aids physiotherapists in promoting sustained physical activity (PA) among patients with (risk of) chronic conditions [10].

To support physiotherapists in their role of health promotor, the *Keep Moving Support Tool (KMST)* was developed, incorporating patients’ perspectives and their wider physical and social context to effectively achieve sustained PA [11, 12]. The KMST was based on behavioural and design-oriented research using the Behaviour Change Wheel [10] (see further details in the methods section). The feasibility of the KMST was evaluated on a small scale with professionals and patients showing promising results [12]. Physiotherapists reported that most parts of the KMST were user-friendly, that the KMST stimulated a person-oriented conversation, and that the conversations provided new insights in the needs and context of the patient leading into customized support of PA behaviour change [12]. Also, preliminary suggestions for future use were mentioned, including simplifying a part of the KMST, integrating the KMST in the electronic patient file, and providing a financial incentive to encourage its use [12].

Currently, while acknowledging above mentioned suggestions, it remains largely uncertain how to effectively integrate the KMST into daily practice of a broader range of primary care physiotherapy clinics in the Netherlands. Health promotion literature so far reported that only a small number of efficacious physical interventions are successfully being used in practice [13, 14]. Moreover, previous studies have demonstrated that implementing person-centered care in physiotherapy clinics, particularly for back pain patients, has been challenging [15, 16]. Physiotherapists generally reported a lack of self-efficacy in person-centered guidance and difficulties in motivating patients to adopt an active lifestyle [16, 17]. Likewise, as our new *KMST* requires a change in workflow and behaviour of professionals, difficulties with correct adoption or implementation of the method in a variety of settings could be expected. Therefore, we considered it crucial to meticulously identify barriers and facilitators during the pre-implementation stage to address them before the actual implementation. The Consolidated Framework for Implementation Research (CFIR) [18] provides a comprehensive and systematic approach for identifying barriers and facilitators for implementation of health interventions. The CFIR is increasingly used in various implementation studies and thereby provides a shared language [19, 20]. The CFIR is derived from a comprehensive review of existing implementation frameworks. It identifies a range of constructs, which include determinants, barriers, and facilitators, that are linked to successful implementation efforts [18]. A recent systematic review that focused on the implementation of PA interventions in physiotherapy clinics identified various common barriers and facilitators using CFIR constructs [21]. These include relative advantage, adaptability, complexity, patient needs and resources, available resources, knowledge and beliefs about the intervention, and self-efficacy [21]. However, the review also revealed significant variation between physiotherapy clinics, highlighting the necessity for a thorough assessment of constructs per clinic. A recent paper on the implementation of the Global Action Plan on Physical Activity stressed the importance of addressing regional, geographic and cultural barriers in the development of advocacy and implementation strategies as well [22]. In addition, previous studies primarily utilized focus groups and/or individual interviews as research methods for this identification process [21]. The current study incorporated an additional, design-oriented research approach—context mapping—to offer a more contextualized needs assessment of professionals within various local primary care physiotherapy clinics [23, 24]. Context mapping enables a deep understanding of the needs of physiotherapists for implementing the KMST in their specific practice settings. Such in-depth insights are essential to tailor the selection of relevant implementation strategies [7, 21].

This research offers a new perspective on planning the implementation of a new PA intervention, using both implementation theory as well as context mapping, to carefully identify barriers and facilitators before implementation occurs. The aim of this study was to develop a generic implementation plan that supports the use of the newly developed KMST in multiple physiotherapy clinics. Specifically, we aimed to (1) identify barriers and facilitators for implementation of the KMST in daily practice; and (2) select implementation strategies and propose corresponding materials and actions to address these barriers and facilitators.

## Methods

**Fig. 1:**
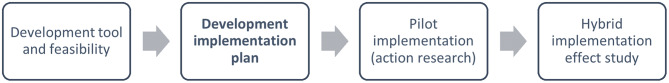
Schematic overview of the overall research project on the development and implementation of the KMST. In bold is the current study

This study is part of a larger research project and serves as a follow-up to a previous study in which the KMST was developed and evaluated [12] (see Fig. 1). Ethical approval was gained from Leiden University of Applied Sciences (METC METC-nr: 19–077). All participants signed informed consent.

### Design

A qualitative design using interviews and context mapping sessions was followed [24]. In addition, a two-step research approach was used [18, 25, 26]. In Step 1, we assessed barriers and facilitators for the implementation of the KMST by conducting semi-structured interviews with experts and context mapping sessions with physiotherapists. Context mapping is a participatory design method that involves mapping the living environment of end-users through generative assignments and intensive contact with them [23, 24]. The goal is to gain deep insight into the environment, as well as the latent needs and feelings of the end-users, enabling them to develop services that match with these needs and feelings [23]. Step 2 comprised of the selection of implementation strategies and their proposed operationalization (materials and actions) to address the barriers and facilitators identified in Step 1.

### Description of KMST

The KMST has been developed to assist physiotherapists in stimulating patient centered support of PA [12]. The KMST comprises five steps: (1) Preparation - gaining insight into the behaviour goal, capability, opportunity and motivation of the patient, (2) the conversation - the patient within his / her context, (3) the intervention decision tool, (4) solutions/interventions, and (5) completion (registration of the agreements) [12]. Several materials have been developed to support these steps, including: six PA profiles that aims to assist the patient with the first step (gaining insight), a decision tool in the shape of a flow chart that supports step 3 and 4 (selecting a(n) intervention(s)), and a manual for the professional and one for the patient. The KMST can be used to prepare for a physiotherapy consult (patients read the PA profiles) and during this consult (the healthcare professional and patient discuss the patient’s needs and a tailored PA intervention is selected using the decision tool).

### Setting

The study was conducted in cooperation with four physiotherapy clinics. Interviews were held online. Context mapping sessions were held at the university campus. Physiotherapists represented four different clinics, that varied in size (6 to 36 physiotherapists). These clinics are situated in various regions in the western and central parts of the Netherlands, encompassing neighborhoods with different socioeconomic statuses. The target patient group for the KMST includes patients with (risk of) chronic conditions (various/unspecified).

### Step 1: needs assessment (barriers and facilitators) for implementation

#### Characteristics of participants

In total, four implementation experts and four physiotherapists participated in this study. The implementation experts were recruited from the network of the authors and partners in the project through purposive sampling. This was done to ensure diversity of expertise and experience regarding implementation in physiotherapy settings. Implementation experts included a senior researcher in implementation science and three innovation and implementation advisors within physiotherapeutic care. The experts were recruited by the project leader (AH) via email or phone. Prior to the interview, a researcher (AH or JD) informed the experts via email about the aim, content and location of the interview, and included an informed consent.

Four physiotherapists, with 2 to 14 years of experience, were recruited from four different practices using purposive sampling from the researchers’ network. Among them, two were practice owners or managers, one specialized in geriatric patients, and one in general physiotherapy. All four physiotherapists worked with patients who have, or are at risk of, chronic conditions. All physiotherapists were involved in the KMST project as partners and have committed to implementing the KMST into their daily practice. Two of them were already familiar with the KMST because of their involvement in the previous development study of the KMST [12]. We selected a small group of four physiotherapists, to facilitate close collaboration in developing an implementation plan tailored to their needs. Previous research has shown that having four to six participants per context mapping session is optimal for fostering a group dynamic, encouraging discussions, and ensuring individual attention [23]. First, the physiotherapists received an email from the project leader (AH) with information about the context mapping sessions. Subsequently, another researcher (JD) provided further details via email about the aims, content and location of the sessions in an information letter and an informed consent form.

#### Interviews and context mapping

Barriers and facilitators for implementation of the KMST were collected in semi-structured interviews with the four implementation experts, and three rounds of context mapping sessions with the four physiotherapists.

The interviews were conducted online by JD, IR, AH and AF, all experienced qualitative researchers, and lasted between 45 and 60 min (October -November 2022). A topic list was developed based on a selection of CFIR domains & constructs in combination with the CFIR interview tool (https://cfirguide.org/tools/). In Table 1 and the additional file 1 the topic list is presented. During a consortium meeting with all interest holders, including physiotherapists, researchers, and representatives of professional associations and patient federations, exploration of important constructs were conducted (see Additional file 2). The selection of CFIR domains and constructs in the topic list was based on results from this meeting. Additionally, the CFIR domains and constructs were aligned with the expertise of the implementation experts. All questions were presented to the three innovation and implementation advisors within physiotherapeutic care. The senior researcher in implementation science) received more general questions about experience with implementation research in the field of physical activity, common barriers and facilitators for implementation and the development of an implementation plan. The interviews were recorded using Microsoft Teams. Transcripts were anonymized and summarized by the researchers independently (JD, IR and AF) and the summaries were discussed and validated by two other researchers independently (SR and AS).

**Table 1:** Topic list interviews (summarized, see also additional file 2)

CFIR domains	CFIR constructs	Topics
Outer setting	Cosmopolitanism	Organisations around physiotherapist Collaborations between organisations and physiotherapists
	External policies and incentives	Measures, policies and regulations or guidelines Financial or other incentives
Process	Engaging	Key influential individuals within organisations
		What is their influence within implementation
Inner setting	Culture	Culture of physiotherapy clinics Norms, values and assumptions How could this culture influence implementation New ideas and innovations within physiotherapy clinics Example of recent situation
	Implementation climate	Implementation of similar interventions Incentives within physiotherapy clinics stimulating implementation Contribution of organisation to development strategy How to involve physiotherapist in the process of implementation

The content of the context mapping sessions was primarily based and organized on the CFIR [18] combined with the COM-B model of the Behaviour Change Wheel [10] (see Table 2). An overview of aims, CFIR constructs, COM-B components, assignments and topics of the three context mapping sessions (Nov 2022 - January 2023) is presented in Table 2. The time between sessions allowed the physiotherapists to consider the barriers and facilitators and steps needed to implement the KMST in their context. In addition, the researchers analyzed the data from previous sessions and carried the data into the next session. For each context mapping session creative assignments were developed that fitted with a selection of the domains and constructs of the CFIR. For instance, all ideas about potential obstacles were collected in a brainstorm exercise and each physiotherapist was asked to draw a patient journey and select time points when the KMST could be used. The selection of domains and constructs was based on experiences with the KMST collected in the development and feasibility study [12]. Additionally, it was based on results of an exploration of important constructs during an interest holders meeting at the start of the project, as well as insights from the interviews. For example, implementation experts emphasized the significance of the perceived value of the new method. Prior to the sessions, sensitizing homework assignments were sent to prepare the physiotherapists for the sessions. The sensitizing assignments were created in such manner to trigger the physiotherapists to contemplate different factors that align with the various constructs as a preparation task.

**Table 2:** Overview of aims, CFIR constructs, COM-B components, assignments and topics of the context mapping sessions

	Session 1	Session 2	Session 3
Aim	Get acquainted with physiotherapists and clinics Map the work process of the physiotherapists Determine how the KMST could fit in the work process	Explore the target group of KMST Explore the connections and interactions within the clinics Determine the most important ‘change agents’ in the clinics	Discuss crucial factors for the implementation of the KMST (how to learn new things) Develop an action plan
CFIR domains: Constructs	Intervention characteristics: Relative advantage Inner setting: Implementation climate (compatibility) Characteristics of individuals: Knowledge & beliefs about the intervention	Inner setting: Networks & communications Process: Engaging (opinion leaders, champions)	Inner setting: Networks & communications, implementation climate (compatibility, organisational incentives & rewards, learning climate) Characteristics of individuals: Self-efficacy, individual stage of change, other personal attributes Process: Planning
COM-B component	Motivation, Capability	Opportunity	Capability, Opportunity, Motivation
Sensitizing assignments (preparation)	Think about a specific situation or experience regarding a change process at work. What went well and what did not?	Keep track of patients you saw and report if you would use the KMST for them. Make a visual overview of your co-workers in our Miro (digital visual workspace for innovation).	Keep track of the contact moments (formal and informal) with co-workers.
Topics and exercises during session	1. Discussion of sensitizing assignment 2. Work processes around PA stimulation 3. Added value of KMST. Chances and obstacles om implementation of KMST in work processes	1. Discussion of sensitizing assignment, who is the patient target group. 2. Connections and interactions with co-workers, who are change agents	1. How to organize learning KMST in practice together with your co-workers. What is needed to feel competent and enthusiastic 2. Initial use of KMST with patients, exchanging experiences with co-workers 3. Creating an action plan regarding learning and using KMST with a small group of change agents

The duration of context mapping sessions were two hours each. They were led by a facilitator (AS), experienced (5 years) in design research, context mapping and co-creation. Two to three other researchers (AF, JD, SR, IR) were present to assist and take notes. All four physiotherapists were present at the first two sessions. Due to personal circumstances, three physiotherapists were present at the final session. The sessions were held in a private classroom and were audio recorded. At the end of each task and before the start of the next context mapping session, the contributions were collectively discussed and summarized, allowing space for additions and feedback. Photos were taken of the completed assignments (for instance, completed work processes and action plans). Using the audio and photos, each session was summarized and anonymized by a researcher, and the summary was complemented and validated by the other researchers that were present (AS, JD, AF, SR, IR).

#### Data analysis

Two researchers (SR and AS) independently coded all data (summaries combined with audio and photos) collected in the interviews and context mapping sessions in Microsoft Excel, using the CFIR constructs in a directed content analysis approach. Directed content analysis is a deductive coding approach and allows the use of existing taxonomies to code data, thereby enhancing the generalizability of results to other contexts [27]. A consensus discussion meeting was held with a third researcher (JD), to solve disagreements and to refine and finalize the coding. Subsequently, one researcher (JD) formulated summary statements inductively per construct and accompanied these summary statements by two exemplary quotes from the data. Another researcher (SR) checked and refined the summary statements.

Based on the constructs and matching summary statements, five overarching themes were constructed by one researcher (JD) that reflected the main needs for implementation. A second researcher (SR) reviewed and refined the themes, which were discussed and finalized in a consensus meeting (AS, SR, JD, AH, AF, IR). The five themes were presented with their corresponding constructs and formed the input for the selection of strategies.

#### Research team and reflexivity

The research team consisted of female researchers with various backgrounds (I.e. human movement science (JD, SdV, PS, MB), behavioral science (SR), health science/physiotherapy (AF, IR), design research (AS), and health science/co-creation (AH) from three Universities of Applied Science (Leiden, The Hague and Rotterdam). All researchers are co-authors of this article. Different researchers (JD, SR, AS, AF, IR and AH, all PhD) were involved in the analyses of which four were not involved in the development of the KMST and several consensus meetings were organized. Involvement of different researchers throughout this process helped attenuate individual biases from the analysis, increased the quality and added credibility to the findings.

### Step 2: selecting implementation strategies

#### Procedure and data-analysis

We selected implementation strategies from the widely used Expert Recommendations for Implementing Change (ERIC) project, in which experts generated consensus on a comprehensive compilation of 73 conceptually distinct strategies [28, 29]. To enhance comprehensiveness in selecting strategies, one researcher (SR) carefully considered each ERIC strategy and coded them according to relevance and feasibility (based on time consumption, finances, alignment with workflow in the clinics). This process was reviewed by another researcher (AH) and subsequently discussed in a consensus meeting with all authors. Irrelevant and/or infeasible constructs were excluded for subsequent selection for the plan. For example, the strategy “use data experts” was considered irrelevant and infeasible for the aim and resources of the clinics. Subsequently, for each theme (and its corresponding constructs) as generated in Step 1 (needs assessment), two researchers (SR and JD) independently selected the remaining relevant strategies for addressing the needs depicted by the theme. Strategies could be applied to more than one theme. A consensus meeting was held to define the final list of implementation strategies and to operationalize these into practical tools. Findings from the interviews and context mapping sessions were used to align the materials and actions with each strategy.

## Results

A list of the collected barriers and facilitators for implementation of the KMST, summary statements, quotations from the interviews and summarized data from the context mapping sessions is presented in Additional file 3. The inventory of barriers and facilitators for implementation of the KMST through interviews with implementation experts and context mapping sessions with physiotherapists were grouped into five themes (Step 1). These themes and matching implementation strategies (Step 2) and materials and actions are presented in Table 3.

**Table 3:** Recognized themes, selected implementation strategies, and suggested materials or actions for implementation

Theme	CFIR Construct	ERIC Implementation Strategy*	Materials/Actions**
1. KMST needs to be understood and appreciated	Evidence strength & quality Relative advantage Complexity Tension for change Relative priority Knowledge & Beliefs about the intervention	Conduct ongoing training Use mass media Facilitation Identify and prepare champions	Training: - E-learning - Workshop behaviour change for physiotherapists Communication: - Promotional Movie - Flyer for patients - Information posters for professional’s treatment rooms - Content for website physiotherapy clinics / narrowcasting Facilitation: - Clinic meetings with core team of early adopters - Support by researchers (action research) Champions: -Assemble core team in each physiotherapy practice
2. Physiotherapist needs to feel and be competent to work with the KMST	Complexity Culture Compatibility Access to knowledge and information (about intervention) Self-efficacy Individual stage of change	Conduct ongoing training Make training dynamic Develop educational materials Distribute educational materials Create a learning collaborative	Training: - E-learning - Workshop behaviour change for physiotherapists Organize learning community
3. The KMST needs to be applied in physiotherapy clinics and implementation needs to be monitored	Adaptability Triability Networks & Communications Implementation Climate Compatibility Goals and feedback Reflecting & Evaluating implementation efforts	Conduct cyclical small tests of change Remind clinicians Promote adaptability Organize clinician implementation team meetings Conduct educational outreach visits Develop and organize quality monitoring systems	Implementation research (Action research) Visits/meetings: - Clinic meetings with core team of early adopters -Visits researchers / implementation expert Monitoring & evaluating: -E-learning -Electronic patient file
4. Several interest holders need to be involved during implementation	Adaptability Patient Needs & Resources Cosmopolitism Leadership engagement Opinion leaders Champions External change agent	Involve patients/consumers and family members Identify early adopters Promote network weaving Build a coalition Inform local opinion leaders	Patients: Feedback + evaluate on method Physiotherapists: Clinic meetings with core team of early adopters External partners: Network building with external partners (GP, sport coaches, professional associations)
5. Resources are necessary to facilitate implementation	Cost External policy & Incentives Available resources Organisational incentives and rewards	Access new funding Fund and contract for the clinical innovation Change accreditation or membership requirements Change record systems	Access new funding -Follow-up implementation research -Reindorsement physiotherapists (health insurance) / financial reward physiotherapists Accreditation Integrate in electronic patient file

* Some ERIC strategies match with more than one construct

** Some Materials/Actions match with more than one ERIC Strategy

### Step 1: barriers and facilitators for implementation

#### Theme 1: KMST needs to be understood and appreciated

Experts and physiotherapists emphasized that enhancing understanding of the relative advantages of the KMST and the strength and quality of the evidence are critical factors for successful implementation. Physiotherapists found the KMST important and described the potential and benefits of the tool.

Context mapping session1b (S1b): *The two physiotherapists that have worked with the KMST believe in the quality of the profiles and their effects.* (Summary statement)

However, they also reported the need to be motivated and know or experience what the added value is of using the KMST compared to usual care. This need for urgency was highlighted by two experts in the interviews. Furthermore, the need for urgency was also mentioned in the first context mapping session a (tension for change & relative priority). If there is no urgency to use the KMST, it could lead to resistance from the physiotherapists.

S1: *There could be rebellion from the clinics. Implementation often comes with resistance. ‘We are going to start something new’*. (Summary statement)

The experts gave suggestions how to create urgency:

*Think about what is the problem that you are solving with the KMST.* (Implementation Expert 1 (IE1))

*What can help in the transition: sense of urgency*,* trust that it could lead to more enjoyable work*,* and being able to better help patients.* (IE3)

Physiotherapists experienced some difficulties in the application (complexity and knowledge & beliefs about the intervention). Choosing the behaviour change techniques matching the needs of their patients was mentioned as the hardest part. Regarding their beliefs about the intervention, one expert said:

*The KMST has to prove itself*,* then you will be willing to use it. If it is something that works*,* then you want to use it.* (IE1)

#### Theme 2: physiotherapists need to feel and be competent to work with the KMST

According to the experts and physiotherapists, behaviour change of the physiotherapist is a crucial part of successful implementation of the KMST. Physiotherapists need to feel competent in applying the new tool. This is viewed as a barrier as adapting to a new way of working is difficult, especially when the identity of physiotherapists is not yet integrated in the new role of health promotor or coach. Hence it also requires a new way of thinking.

*The role of ‘health promotor’ – coaches and physiotherapists need to get used to this and regard it as part of their work* (IE1)

Also, as indicated before, the KMST itself is viewed as difficult by the physiotherapists, especially the part where they need to select a behaviour change technique intervention. Physiotherapists may be in different phases of behaviour change themselves with respect to the tool. In general, they do not feel competent in using the tool, and some may even feel resistance. Therefore, it is important to assess for each physiotherapist where they stand with respect to feeling competent and motivated in applying the tool. Physiotherapists indicated various strategies that may help them, and future users feel enthusiastic about the tool:

S3: *Knowing the motivation behind KMST*,* success stories*,* learning together with colleagues*,* reminders to keep using the KMST and perhaps a reward system.* (Summary statement)

#### Theme 3: the KMST needs to be applied in physiotherapy clinics and implementation needs to be monitored

To be able to learn from and get acquainted with the KMST the experts found it necessary to stimulate physiotherapists to start using the KMST. Experts advised to start on small scale, to monitor that, ask for feedback and adjust along the way:

*At the start potentially also communicate extensively*,* be in contact… Ask for feedback*,* if it does not work*,* make adjustments to that.* (IE3)

According to the physiotherapists, it is important to organize a pilot and to schedule meetings with physiotherapists, for instance a lunch break, weekly meetings or practice meetings. These meetings can be used to monitor and discuss the use of the KMST. Evaluation of the KMST by the physiotherapist and patient is important as well.

S2: *In clinics*,* different moments are suitable for consultation/feedback (lunch*,* weekly meetings*,* practice meetings plus lunch)* (Summary statement).

Adaptability of the KMST was mentioned by the experts regarding the use for different target groups. A facilitator for implementation would be adjusting the implementation strategies to the physiotherapy clinics’ needs. In addition, the experts highlighted that the implementation strategies need to be evaluated.

*Ask clinics (physiotherapist and patient) if the implementation strategies are experienced as ‘supportive’; what was difficult and what went well?* (IE2)

#### Theme 4: several interest holders need to be involved during implementation

From the interviews it became clear that various interest holders should be considered for successful implementation, both within and outside the clinics. Within the clinics, it is important to recognize employees that are early adopters, who are dedicated to taking up and driving the implementation, such as practice owners and physiotherapists who are open for training.

*First focus on early adoptors*,* the practice owners*,* they are motivated the most.* (IE1)

The wide variety of patients suitable to work with the KMST are also important to consider. For certain target groups the KMST may be less suitable, such as patients with lower health literacy. For other specific target groups the KMST could be used after a certain triage.

*KMST could be applied after a certain triage (a few questions) for a specific target group. For each target group you could expand existing programs with KMST (*IE3)

Outside the clinics, there are important interest holders that need to be considered for collaboration. A sports coach, general practitioner or neighbourhood team may stimulate the referral of patients. Also, other regional partners or multidisciplinary collaborations in primary care are important for wider implementation. Experts considered it also helpful to have an ambassador, an individual with authority who acts as an advocate for the new KMST, which could be a physiotherapist or a physician.

*Ambassadors are important. Someone with authority*,* who acts as an advocate. Often a physician*,* but it could be a physiotherapist as well.* (IE3)

#### Theme 5: resources are necessary to facilitate implementation

Experts and physiotherapists indicated that a lack of time and funding could be an obstacle in the implementation of the KMST. Physiotherapists were also afraid that it might take them more time than expected to get acquainted with the KMST and to use it with their patients.

S1: *Consults cost money*,* KMST may require more than 1 consult. This will be subtracted from the consults paid by health insurance. What about the funding for that?* (Summary statement)

Facilitators mentioned were accreditation for the physiotherapists and integration of the KMST in the electronic patient file used by the physiotherapy clinics, which could enhance documentation.

A sufficient reward for the physiotherapists could stimulate the use of the KMST.

S3: *Physiotherapists are excited about a reward system with colleagues; The question was asked whether accreditation or external rewards are feasible options.* (Summary statement)

### Step 2 selecting implementation strategies

For each construct implementation strategies were selected matching with the barriers and facilitators extracted from the data. Based on these strategies, materials have been proposed, or already developed or upgraded, and actions necessary for implementation of the tool have been formulated (see Table 3). We highlight a few materials and actions here.

Communication materials are needed to inform physiotherapists and patients about the KMST in general, the added value and the strength of the evidence for its added value (theme 1). In order to increase the knowledge of the physiotherapists of the KMST and increase competencies in using the method, training of the physiotherapists is needed. Therefore, an e-learning was developed as a result of this study by the researchers and tested by physiotherapists during a pilot action research study. The e-learning was a training platform (contained background information, explanation of the KMST, and case studies on how to apply the KMST in clinical practice), which was hosted on a software platform used for training purposes. Developing a clinic-tailored action plan including meetings with physiotherapists is part of the e-learning as well.

Next to that, a workshop for the physiotherapists has been developed with the aim of assisting them in the process of changing behaviour of their patients and to share experiences with their peers. A learning community with physiotherapists can help increase their competencies by offering a platform to discuss lessons learned in implementing the KMST.

It is important to assemble a core team of early adopters (identify champions) per practice and to organize meetings with this group (theme 1, 3 and 4). This way, physiotherapists can be facilitated in the application of the tool and share experiences, what could help to increase appreciation, understanding and could be a start of a network in the clinics. Support from researchers (including visits) as part of an action research could also facilitate the physiotherapists.

Patients, physiotherapists and interest holders need to be involved in implementation (theme 3). Experiences of patients with the KMST could be collected and the effect of the KMST on their PA behaviour needs to be evaluated as part of action research. Physiotherapists can exchange experiences in meetings with their core team of early adopters. In addition, a network with other interest holders should be built in order to improve the patient’s journey from guided PA in healthcare setting to PA at home (theme 4).

Regarding resources (theme 5), a financial reward or sufficient reindorsement for supporting PA during a consultation and accreditation could stimulate physiotherapists to apply the tool.

## Discussion

In this study we identified barriers and facilitators for implementation of the KMST in daily physiotherapy practice and selected matching implementation strategies. Using the evidence-based CFIR [18], the combined data from the interviews with implementation experts and context mapping sessions with physiotherapists helped to elucidate essential aspects that need to be addressed to increase the successfulness of uptake and use of the new method in multiple physiotherapy clinics. The suggested implementation plan consists of a relevance- and feasibility-based selection of ERIC strategies [28] and corresponding materials and actions. These include communication (promotion) materials, assembling a core team of implementation champions per clinic, training, facilitation through consultation meetings and outreach visits, a learning community, and adjustments to the electronic patient file. Monitoring and evaluation of the uptake of KMST was also listed as a relevant strategy, which is part of the subsequent action research, during which proposed materials and actions are being implemented and adjusted to the specific context of the clinics.

This research shows a novel approach to structurally develop an implementation plan. Next to consulting implementation experts through interviews, we employed a context mapping approach that has its foundation in design research and is new to healthcare implementation research [23, 24]. We advise future research in implementation to consider this participatory, context-based approach in the development of an implementation plan. A second novelty was that the development was planned in the pre-implementation phase, as the intervention itself (the KMST) was newly developed and had yet to be adopted by physiotherapists. A systematic review on the use of CFIR in implementation showed that most studies conducted a post-hoc analysis on what had facilitated or hindered implementation of an intervention already in use [30]. The identification of barriers and facilitators that could affect subsequent implementation in the pre-implementation phase has rarely been done, and could be a new way of applying implementation frameworks and add to previous research on the application of implementation frameworks [31]. We consider it a particular strength that we developed an implementation plan prior to the first use of the intervention, for several reasons. First, identified barriers can be addressed at the earliest stage, prior to actual implementation. Second, it increases the likelihood that the end users (physiotherapists) will use the new method as intended (i.e. a higher fidelity), while allowing room for intervention adjustments so that it better aligns with the end-user and its context. Indeed, adjustments have been planned, including the creation of an additional implementation tool to simplify the selection of the interventions. Third, involving the end user from the start and thereby co-creating the implementation may create a sense of psychological ownership, which enhances valuation of the created ‘product’ [32].

Overall, the use of the CFIR in our needs assessment led to the identification of a wide variety of barriers and facilitators, and hence a wide variety of implementation strategies. Some strategies served multiple barriers and facilitators. Many of these strategies (materials and actions) serve to increase physiotherapists’ competency (self-efficacy) of applying the KMST (i.e. e-learning, workshops, consultation meetings, learning community and outreach visits). This may not be surprising, as our new tool requires a new way of working including shared decision making, motivational interviewing skills and the selection of behaviour change strategies. Previous research also showed that a training in person-centered care among physiotherapists was experienced as (amongst others) learning a new language and led to feelings of overwhelm and discomfort [17]. A recent report of the WHO stressed the importance of supporting health, sport, and exercise professionals in the implementation of strategies [33]. Our multifaceted approach addressing competency seems to be adequate, since several systematic reviews showed that multifaceted educational interventions are most effective in implementation of clinical guidelines among healthcare professionals, including physiotherapists [34–36].

Based on our current findings, future implementation endeavors include providing accreditation for completing the e-learning and building a network with external partners. Further research is planned to evaluate the implementation materials and actions, and to measure the effectiveness of the *KMST* in stimulating sustained physical activity in patients with chronic conditions. In addition, a future step is to make the KMST accessible to a broader range of patients.

Besides focusing on the pre-implementation phase, this study has other strengths. First, the use of theoretical frameworks, primarily the CFIR and secondarily the Behaviour Change Wheel/COM-B, ensured an evidence-based structured approach to identify relevant barriers and facilitators. Two recent reviews indicated that most implementation studies did not report use of a theoretical framework to inform data collection [21, 30]. Second, the variety of involved clinics and physiotherapists, in terms of patient population, years of experience, and size of the clinics increases the applicability of results to other, future clinics interested in implementing the new method.

Some limitations need to be considered when interpreting our findings. First, physiotherapists were self-selected to participate in this study, thereby representing a sample of individuals motivated to adopt and implement a new tool to support physical activity. This selection method could have resulted in selection bias. Yet, we expect that future implementation will also engage physiotherapists with a particular interest in this new tool. Second, the included clinics were situated in an urban area in the Western part of the Netherlands, which limits generalizability of findings to other contexts, especially since the tool promotes context-based PA. It remains unclear how the new tool would be perceived in a different (rural) area or country. Third, the coupling of ERIC strategies to the CFIR constructs was largely based on the judgement of researchers. A suggestion for future research is to select ERIC strategies in co-creation with participants. Nevertheless, researchers were present during the context mapping during which not only needs were collected, but also possible solutions were presented by physiotherapists. A so called “matching tool” exists for the purpose of matching CFIR constructs and ERIC strategies, yet the reliability of this tool is questionable, given that implementation experts showed considerable heterogeneity in opinions of what strategies are best for a given construct (barrier) feasibility in terms of time and finances [37].

## Conclusion

Our results from interviews and context mapping sessions, guided by the CFIR, revealed essential aspects that need to be addressed in the pre-implementation phase to increase the successfulness of implementation of the KMST in daily practice of physiotherapy clinics. Among a wide variety of barriers and facilitators for implementation, we found that implementation efforts should first and foremost be focused on increasing physiotherapists’ competency in using the KMST. Our generic implementation plan intends to support physiotherapists among multiple clinics that are in the challenging need to provide person-centered guidance and self-management of sustained PA of patients with chronic conditions.

## Supplementary Information

Below is the link to the electronic supplementary material.

Supplementary Material 1

Supplementary Material 2

## Data Availability

Summarised data analysed for this study are included within the text, tables and additional files of this article. The raw dataset generated and analysed during the current study is not publicly available as no statement regarding the publication of qualitative data was included in the informed consent.

## Abbreviations

ERIC: Expert Recommendations for Implementing Change
CFIR: Consolidated Framework for Implementation Research
IE: Implementation expert
KMST: Keep Moving Support Tool
PA: Physical activity
S: Session (context mapping)
