# Supplementary material for: Using context mapping for planning implementation of movement behavior change in physiotherapy
Source: BMC Health Serv Res. 2025 Dec 22;26:118. doi: 10.1186/s12913-025-13897-x (PMC12838495; doi:10.1186/s12913-025-13897-x)
Supplement: Supplementary file 1 — Supplementary Material 1 [file 12913_2025_13897_MOESM1_ESM.docx]

**Additional files**

**Additional file 1: Topic list interviews**

***Main question: Based on CFIR, What are promoting and hindering factors?***

OUTER SETTING

*We start with important organisations or networks that a physiotherapist has to deal with during the implementation of a new intervention.*

Cosmopolitanism

1. Which organisations, agencies, networks, and systems are important around the physios when implementing a new intervention?
2. What do these organisations, agencies, networks do? (*connection between community sports coaches / exercise coaches / IOF / chronic network?)*
3. What rules, regulations, policies, and/or guidelines are attached to it?
4. What does that mean for the physios?
5. What are the collaborations between the above-mentioned organisations/agencies/networks and the physios?
6. What is the (desired) involvement?
7. What is exchanged? information, people, technology, rules, etc.
8. What exchange structures are there? formal, informal.

PROCESS

Engaging

1. Who are the 'key influential individuals' within these (above-mentioned) parties?
2. Have these people been formally put in this position or is it informal influence?
3. What are these people important for? What influence/role do they have within the implementation process?

OUTER SETTING

*We will now discuss important guidelines or measures or incentives that may affect the process of implementation.*

External Policies and Incentives

1. What local, regional, or national measures, policies, regulations, or guidelines might influence the implementation of an intervention? (promote / hinder)
2. What kind of financial or other incentives could positively or negatively influence the implementation of an intervention like Keep Moving Support Tool? (*possible questions on EPD and method of funding*)
3. What kind of measures/incentives do you offer?

*(Zoom in on physio practice)*

INNER SETTING

*The last questions are about the setting of the physiotherapy practice. What does the culture look like and the implementation climate?*

Culture

1. In general, how would you describe the culture of physiotherapy clinics?
2. Norms, values, assumptions that people have
3. How could this culture influence the implementation of Keep Moving Support Tool?
4. To what extent are new ideas and innovations embraced within physiotherapy clinics? (positive and negative)
5. Can you give an example of a recent situation?

Implementation climate

1. How have similar interventions been received by physiotherapy clinics?
2. Are there certain incentives within physiotherapy clinics that can stimulate the implementation of interventions?
3. How could your organisation contribute to the development strategy?
4. How do you involve the physiotherapist in the process of implementing interventions? (*training/ facilitation/ how to connect with exercise coaches?*)

**Concluding question**:

What is the most important thing you would like to give us for a successful implementation of the Keep Moving Support Tool method?

**Additional file 2**

Table 3. Barriers and facilitators for implementation of the Keep Moving Support Tool

|  | **Facilitator or potential barrier** | **Summary statement** | **Quotations and summarised data ^a^** | **Quotations and summarised data ^a^** |  |
| --- | --- | --- | --- | --- | --- |
| **CFIR Domain I: intervention characteristics** |  | | | |  |
| B Evidence strength & Quality | Facilitator | Evidence strength (effectiveness) and quality of care are important for the implementation. | Evidence strength is of importance, it needs to be effective care. That is why it often takes quite some time before it is used in practice. (IE1^c^) | S1^b^: The two physiotherapist that have worked with Keep Moving Support Tool believe in the quality of the profiles and its potential impact. |  |
| C Relative advantage | Facilitator | Physiotherapists see added value or potential for the Keep Moving Support Tool method, but need to be motivated and see what it could bring them before they are going to use it. | You constantly need to show the value and motivate them.  Indicate the chances it offers and for whom. (IE1) | S1: added value was expressed in many ways, e.g. steering conversation between therapist and patient, guideline for all therapists, get patients passionate about physical activity. |  |
| D Adaptibility | Facilitator | The Keep Moving Support Tool method can be used for specific target groups. For the implementation: adjust to the needs from the physiotherapy clinics. | Differences between clinics, toolbox with strategies from which clinics can choose. (IE2) | Keep Moving Support Tool could be applied after a certain triage (a few questions) for a specific target group. For each target group you could expand existing programs with Keep Moving Support Tool. |  |
| E Triability | Facilitator | First, start small, ask for feedback and adjust. | At the start potentially also communicate extensively, be in contact... Ask for feedback, if it does not work, make adjustments to that.  (IE3) | You could at first see how it works with the individual physiotherapists.  (IE2) |  |
| F Complexity | Potential barrier | The Keep Moving Support Tool method is experienced as difficult. Behaviour change of the physiotherapist is difficult. | Behaviour change of the physiotherapist is more difficult / takes more energy than expected. (IE3) | S1: The method is difficult; flow chart is not optimal; last part of method, choosing the behaviour change technique is the hardest part. |  |
| H Cost | Facilitator and barrier | Financing is a barrier, while recurrence guarantee is a facilitator. | S1: Consults cost money, Keep Moving Support Tool may require more than 1 consult. This will be subtracted from the consults paid by health insurance...What about the funding for that? | A benefit for the patient is the recurrence guarantee. (IE3) |  |
| **CFIR Domain II: Outer setting** |  | | | |  |
| A Patient Needs & Resources | Potential barrier | For certain target groups Keep Moving Support Tool is less suitable; there is a need for an easier flow chart for patients. | S1: It is difficult for patients with reduced health literacy. Does the patient understand it? | S2: For patients with a language barrier, dementia, etcetera difficult to use Keep Moving Support Tool. |  |
| B Cosmopolitism | Facilitator | Collaboration with important stakeholders is needed to stimulate referral of patients and set up collaboration in regions. | Refer to for instance a sports coach. Role of gatekeeper to secondary care. (IE1) | Motivated general practitioner can facilitate. Sport clubs or a sports coach should be aware of Keep Moving Support Tool. This could initiate a flow of patients. (IE3) |  |
| D External policy & Incentives | Facilitator | Enabling factors are accreditation, including Keep Moving Support Tool in electronic patient files and financing. | S1: Keep Moving Support Tool should be in electronic patient files. S3: Maybe incorporating Keep Moving Support Tool education in accreditation? | Financing is important. There will be more room for this in health insurance policy. (IE3) |  |
| **CFIR Domain III: Inner setting** |  | | | |  |
| B Networks & Communications | Facilitator | Consultation structure is available to discuss Keep Moving Support Tool; just like networks in clinics. | S2: In clinics, different moments are suitable for consultation/feedback (lunch, weekly meetings, practice meetings plus lunch). | Regularly contact with practice owner and quality manager about implementation and monitoring. (IE3) |  |
| C Culture | Potential barrier | The role of physiotherapist is changing, this is hard for some physiotherapists. | The role of ‘health promotor’ – coach, physiotherapists need to get used to this and regard it as part of their work. (IE1) | Identity must change, because the society asks for it. This is very difficult for some physiotherapists. (IE1) |  |
| D Implementation Climate |  |  |  |  |  |
| 1 Tension for change | Potential barrier | There needs to be an urgency, if not, it could lead to resistance. | If the urgency is not clear, there may be resistance. (IE1) | S1: There could be rebellion from the clinics. Implementation often comes with resistance. ‘We are going to start something new’. |  |
| 2 Compatibility | Potential barrier | Using Keep Moving Support Tool asks for a new way of thinking. Also it is important to plan the use of it. | Physiotherapist will be placed in a less executive role and a more supporting role. This asks for a new way of thinking. (IE1) | You need to align with the practice: what is going on that makes it difficult. (IE2) |  |
| 3 Relative priority | Facilitator | It is important to create urgency. | What can help in the transition: sense of urgency, trust that it could lead to more enjoyable work, and being able to better help patients. (IE3) | Think about what is the problem that you are solving with Keep Moving Support Tool. And if you connect to that, then they will be triggered to use it. That is creating urgency. (IE1) |  |
| 4 Organisational incentives and rewards | Facilitator | A reward system should stimulate the use of Keep Moving Support Tool. | S3: Physiotherapists are excited about a reward system with colleagues; The question was asked whether accreditation or external rewards are feasible options. | Financial reward works for simple tasks. For more complicated tasks, it helps more to offer them a podium for appreciation. (IE1) |  |
| 5 Goals and feedback | Facilitator | Evaluation of the tool by the physiotherapist and patient is important. | Provide feedback on the use of Keep Moving Support Tool, plan these meetings and integrate these in the agenda. (IE1) | S3: Important to involve patients: together embark on a process and evaluate the method. |  |
| 1 Leadership engagement | Facilitator | Using an ambassador is an enabling factor. | Ambassadors are important. Someone with authority, who acts as an advocate. Often a physician, but it could be a physiotherapist as well. (IE3) |  |  |
| 2 Available resources | Potential barrier | A lack of time and money could be an obstacle. | Time and money are potential obstacles. (S1) Through financing, you create more room for development. (IE1) | In a previous project obstacles for implementation were the longer intake and needing more training. (IE3) |  |
| 3 Access to knowledge and information (about intervention) | Facilitator | There is a need for training and intervision. Tools for informing the physiotherapists is important. | You need good tools to be able to inform in the right way. (IE3) | S3: physiotherapists want to learn together. Evaluate, tips and tricks via e-learning, a learning community, extra training for the flow chart to choose interventions. |  |
| **CFIR Domain IV: Characteristics of individuals** |  | | | |  |
| A Knowledge & Beliefs about the intervention |  | Physiotherapists find the Keep Moving Support Tool method important, but hard to use. It needs to prove itself. | Keep Moving Support Tool has to prove itself, then you will be willing to use it. If it is something that works, then you want to use it. (IE1) | S3: Physiotherapists see Keep Moving Support Tool as important, but also as something that is not easily used. |  |
| B Self-efficacy | Potential barrier | Physiotherapists do not feel competent in using Keep Moving Support Tool, and they might experience fear. | Physiotherapists need to gain competency in other way of working with the patient. (IE2) | S3: Physiotherapists feel incompetent in motivational interviewing, behaviour change, use of flowchart. |  |
| C Individual stage of change | Potential barrier | Behaviour change is difficult for some physiotherapists, important to assess the phases of behaviour change. Important to motivate and remind them of using the tool. | Resistance is usually not present in the whole group, but a few employees. They are in different phases of behaviour change. Assess for each employee, where do you stand, what questions do you have? (IE1) | S3: What physiotherapists need to feel enthusiastic about Keep Moving Support Tool: knowing the motivation behind Keep Moving Support Tool, success stories, learning together with colleagues, reminders to keep using the tool. Perhaps a reward system. |  |
| **CFIR Domain V: Process** |  | | | |  |
| B Engaging |  |  |  |  |  |
| 1 Opinion leaders | Facilitator | Early adopters and ambassadors are important. | First focus on early adopters, the practice owners, they are motivated the most. (IE1) | Ambassadors are important. Someone with authority, who acts as an advocate. Often a physician, but it could be a physiotherapist as well. (IE3) |  |
| 3 Champions | Facilitator | Physiotherapists that may act as champions are often the practice owners, people who follow training or train others. | S2: It was discussed who are open to change in the clinics: young people, owners, people who follow training or who train others. |  |  |
| 4 External change agent | Facilitator | Regional and multidisciplinary collaborations are of importance. | (Social) neighbourhood teams could have a signaling role. (IE3) | Regional partnerships and multidisciplinary collaboration in primary care are encouraged. (IE3) |  |
| D Reflecting & Evaluating implementation efforts | Facilitator | Implementation research is important. | Evidence of the quality of care is important, treatment numbers, content of care. (IE1) | Ask clinics (physiotherapist and patient) if the implementation strategies are experienced as ‘supportive’; what was difficult and what went well? (IE2) |  |

**^a^** Quotations were shortened and modified to exclude superfluous text yet are intended to represent an accurate reproduction of the data. The context mapping data consisted of verbatim text in a group setting, as well as filled in (written) assignments: data were merged and summarized to indicate a particular structure in a theme [30]
^b^ Session number context mapping
^c^ Implementation Expert number
